# Dopaminergic and noradrenergic manipulation of anticipatory reward and probability event-related potentials

**DOI:** 10.1007/s00213-020-05515-x

**Published:** 2020-04-20

**Authors:** Iris Schutte, Peter K. H. Deschamps, Peter N. van Harten, J. Leon Kenemans

**Affiliations:** 1 Department of Experimental Psychology, Helmholtz Institute, Utrecht University, Heidelberglaan 1, 3584 CS Utrecht, The Netherlands; 2 Department of Psychiatry, Brain Center Rudolf Magnus, University Medical Center Utrecht, Utrecht, The Netherlands; 3 Department of Psychiatry and Psychology, Maastricht University Medical Center, Maastricht, The Netherlands; 4 Psychiatric Center GGZ Centraal, Amersfoort, The Netherlands

**Keywords:** Reward, Probability, Event-related potentials, Reward positivity, P300, Haloperidol, Clonidine, Dopamine, Norepinephrine

## Abstract

Predicting what will happen in the future in terms of potential reward is essential in daily life. The aim of the current study was to investigate the neurotransmitter systems involved in the anticipation of reward value and probability. We hypothesized that dopaminergic and noradrenergic antagonism would affect anticipation of reward value and probability, respectively. Twenty-three healthy participants were included in a haloperidol (2 mg) × clonidine (0.150 mg) × placebo cross-over design and subjected to a Go/NoGo experimental task during which cues signaled the probability of subsequent target appearance. Reward value (amount of money that could be won for correct and fast responding to the target) as well as probability of target appearance was orthogonally manipulated across four task blocks. Cue-elicited EEG event-related potentials were recorded to assess anticipation of value and probability, respectively. The processing of reward value was affected by dopaminergic antagonism (haloperidol), as evidenced by reduction of the reward-related positivity and P300 to reward cues. This reduction was specifically significant for subjects with high baseline dopamine levels for the P300 and most pronounced for these subjects for the reward-related positivity. In contrast, the processing of reward probability was affected by noradrenergic antagonism (clonidine). In addition, both drugs reduced overall performance (omission rate, response speed variability). We conclude that at least anticipation of reward value and probability, respectively, is specifically affected by dopaminergic versus noradrenergic antagonism.

**Electronic supplementary material:**

The online version of this article (10.1007/s00213-020-05515-x) contains supplementary material, which is available to authorized users.

## Introduction

Predicting future reward value and the likelihood of prospective outcomes enables one to select appropriate actions that maximize gain (Glimcher and Rustichini 2004). Clarification of the brain mechanisms of decision-making as driven by cues signaling reward value and probability is relevant to our understanding of various psychiatric disorders associated with impairments of reward-related decision-making (e.g., depression, ADHD, schizophrenia, addiction).

Prior studies have shown that manipulations of anticipated reward value affect a frontal event-related potential (ERP) early in time (“reward-related positivity” (RRP); Doñamayor et al. 2012; Flores et al. 2015; Holroyd et al. 2011; Krigolson et al. 2014; Schutte et al. 2019) and a more parietal ERP later in time (“reward P300”; Broyd et al. 2012; Flores et al. 2015; Pfabigan et al. 2014; Schutte et al. 2019). Anticipation of the probability of (rewarded) targets elicited a separable frontal ERP component (“probability-related positivity” (PRP); Schutte et al. 2019). Here, we aimed to investigate whether these reward and probability ERPs can be dissociated in terms of underlying neurotransmitter systems. Specifically, we tested the hypothesis that, respectively, dopamine (DA) and noradrenaline (NA) are involved in generating these ERPs.

Informative reward cues cause abrupt (phasic) changes in DA level, which have been hypothesized to signal deviations in expected reward value and are associated with immediate enhancements in motivation and/or task engagement (Collins and Frank 2016; Hamid et al. 2016). These DA signals are thought to depend on subcortical transient DA increases that have been related to cortical action, i.e., the effect of DA fluctuations on cortical mechanisms underlying subsequent action to an imperative stimulus (Frank and O'reilly 2006). The present study addresses the sensitivity of these cortical mechanisms, including RRP and P300, to DA and NA manipulations.

Dopaminergic drugs and their effects on (reward) prediction ERPs have so far only been studied in experiments that focused on post-reward mechanisms. These concern the error-related negativity (ERN) and the feedback-related negativity (FRN). Both can be conceived as quick and transient negative reward prediction errors, presumably driven by phasic dips in DA release after omissions of (highly) expected rewards. The role of phasic dips in DA release after unexpected reward omission is supported by studies showing that DA agonist treatment leads to increased amplitudes of the ERN or FRN (ERN, Barnes et al. 2014; De Bruijn et al. 2004; De Bruijn et al. 2005; Spronk et al. 2016; FRN, Santesso et al. 2009), whereas DA antagonists had the opposite effect (2.5 and 3 mg haloperidol, ERN; De Bruijn et al. 2006; Forster et al. 2017; Zirnheld et al. 2004).

Prior studies (Cools et al. 2009; Frank and O'reilly 2006) have demonstrated that (the effect of DA drugs on) reward-based learning, which is thought to be dependent on these reward prediction ERPs is modulated by baseline DA functioning. Cools and colleagues (Cools et al. 2009) found that the DA agonist bromocriptine enhanced reward-based reversal learning in subjects with low baseline DA, while it had the opposite effect in subjects with high baseline DA. Previous work has identified a genotype dependence of D2-antagonist effects on reward outcome–related ERPs (Mueller et al. 2014). Evidence for the relationship between a more endophenotypic index of baseline DA (eye blink rate (EBR)) and the effect of DA drugs on reward anticipation–related ERPs and concomitant reward-based decision-making however is lacking. The current study aims to shed light on this relationship, which will have important implications for our understanding of the strong individual differences in DA drug efficacy seen in the clinic.

The NA system may have a complementary role in reward processing. NA firing is associated with the amount of effort needed to obtain a reward only *after* the initial choice has been made to work for the reward (the latter was modulated by DA) (Floresco 2015; Varazzani et al. 2015). Therefore, it may be hypothesized that after initial DA–based estimation of future reward value, perhaps aiding the decision to work hard or not, the NA system may be activated in order to mobilize cognitive and physical resources accordingly (Floresco 2015).

In our prior study (Schutte et al. 2019), cues signaling a high probability subsequent target elicited a frontal-central positivity ERP. It should be noted that this probability-related positivity did not resemble the more posteriorly distributed P300, which has been reported as sensitive to the probability of events and hypothesized to index activity of the NA system (Bekker et al. 2004; Duncan-Johnson and Donchin 1982; Joseph and Sitaram 1989; Nieuwenhuis et al. 2005). However, there are strong indications that the P300 in the context of reward processing may be particularly dependent on DA (Pfabigan et al. 2014). In the current study, we investigated whether the PRP and reward P300 are dissociable in terms of underlying neurotransmitter systems.

In sum, we aimed to uncover the roles of the DA and NA system in modulating cortical mechanisms activated in the context of reward anticipation. This was investigated by blocking the DA and NA system, respectively, by 2 mg haloperidol and 0.150 mg clonidine. We furthermore investigated whether these modulations were accompanied by a concomitant performance decrease. Another main aim was to investigate whether baseline DA modulates the effect of DA drugs on reward-related ERPs. We expected the reward P300 and RRP to be reduced under haloperidol and the PRP to be reduced under clonidine. A placebo-controlled cross-over study was performed using the cued Go/NoGo task from our prior study (Schutte et al. 2019) in which cues predicted upcoming targets with varying reward value and probabilities. Spontaneous EBR was used as an indirect measure of baseline striatal dopaminergic activity (Jongkees and Colzato 2016). We expected the attenuating effects of haloperidol on reward-related ERPs to be more pronounced in subjects with high EBR (Cools et al. 2009). Finally, to further account for individual differences in drug responsiveness, we compared the effects of clonidine and haloperidol on reward- and probability-related ERPs between drug responders and non-responders (see “Methods” section).

## Methods

### Subjects

Twenty-nine healthy males were enrolled in the study (sample size justification can be found in supplementary materials, section 1). Subjects were recruited via advertisement at the Utrecht University and via a recruitment website. Exclusion criteria were (1) a history of relevant medical conditions or mental health issues, (2) current medication use, (3) smoking, (4) a history of cocaine use, (5) daily consumption of > 3 standard alcoholic beverages, (6) more than one occasion of recreational drug use per month, and (7) low blood pressure (< 90 mmHg systolic and/or < 60 mmHg diastolic), low or high heart rate (< 55 or > 100 bpm) during first study visit.

Participants were requested to abstain from consuming xanthines and alcohol for at least 12 h prior to each session, and to refrain from psychotropic drugs for at least two weeks prior to each session. All participants declared to have normal or corrected to normal vision. The study was approved by the medical ethical committee of the University Medical Center Utrecht and conducted in accordance with the Declaration of Helsinki. The study was pre-registered in the Netherlands Trial Registry (NTR), number NTR5019. Participants received 10 Euros per hour and could win a maximum of 15 Euros per session during the cued Go/NoGo task.

Two subjects were excluded because of voluntary withdrawal and one because of nausea during the placebo session. Data of three subjects were discarded during the analyses (see “Data reduction and analysis” section). The final sample consisted of twenty-three males (mean age (± sd) 22.8 (± 3.7) years).

### Pharmacological manipulation

Haloperidol 2 mg is a potent antagonist of the dopamine D2 receptor and is assumed to attenuate neurotransmission in the mesocortical and mesolimbic DA pathway (Kapur et al. 2000). Clonidine 0.150 mg binds to pre-synaptic α2-adrenergic receptors, which has an inhibiting effect on NA release from broadly distributed NA nerve terminals (Svensson et al. 1975). All capsules were over-encapsulated by the pharmacy to ensure double-blinding.

### Cued Go/NoGo task

**Fig. 1:**
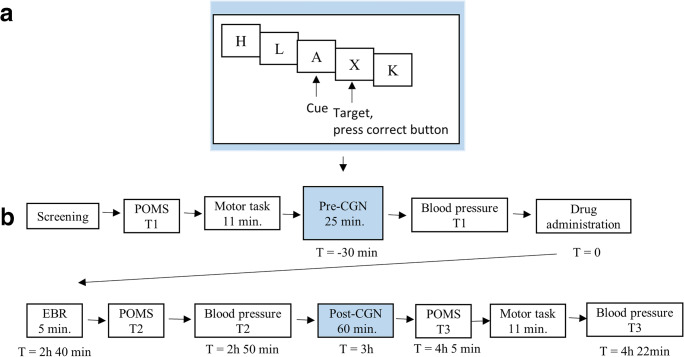
Procedure and cued Go/NoGo task. (A) Participants were subjected to the cued Go/NoGo task twice per session, i.e., to a short version before drug administration and to a longer version with EEG recording 3 h after drug administration. Subjects wore an Actigraph around the right ankle during both cued Go/NoGo task versions. Subjects sat in a comfortable dentist chair 1 m in front of a computer screen in a separate dimly lit room. The subject’s eyes were at the level of the center of the screen. Subjects were instructed to press a pre-specified button when letter X or Y followed the cue (always letter A). The amount of money for correct and fast responding and the probability that a cue would be followed by a target were orthogonally manipulated across four task blocks. The amount of money was either 0 or 5 Euros maximally per reward block (or 0 and 2.5 Euros during the pre-drug task version), and the probability of target appearance was either 50% or 98%. (B) Overview of the procedure of one test session of the main study. The main study consisted of three sessions separated by at least one week. During each session, subjects received 0.150 mg clonidine, 2 mg haloperidol, or placebo. Session order was balanced across subjects

The same cued Go/NoGo task was used as in Schutte et al. (2019). The task is illustrated in Fig. 1A. Details of the task can be found in the supplementary materials, section 2. Subjects had to press a left or right button when a target letter (letter X or Y) followed a cue letter (always letter A), as fast and accurately as possible.

The amount of money that could be won for correct and fast responses (a total of either 0 Euros or 5 Euros during the block) and the probability of target appearance after the cue (either 50% or 98%) were orthogonally manipulated across four task blocks. This reward value and probability information was shown to participants at the beginning of each block. During reward blocks of the pre-drug task version, which lasted half as long, 2.50 Euros could be won.

Participants were assigned to one of four possible task block orders. For each participant, this order was kept the same for the pre- and post-version of the cued Go/NoGo task and for all three sessions. Task block order was counterbalanced across participants and drug order.

### EEG-EOG data acquisition

The electroencephalogram (EEG) and electrooculogram (EOG) were recorded with the ActiveTwo system (Biosemi, Amsterdam, The Netherlands) using 64 Ag-AgCl electrodes placed according to the international 10/10 system. The horizontal and vertical EOG were recorded from electrodes placed above and below the left eye and electrodes at the outer canthi of both eyes. Signals were online referenced to the Common Mode Sense/Driven Right Leg electrodes and online filtered with a filter at DC to 400 Hz (default); the sample rate was 2048 Hz.

Eye blink data (EOG) were recorded, while subjects looked at a fixation cross on the screen (white cross on a black background) for five minutes. Subjects received the following instructions: “We will now proceed with a measurement during rest. Please sit comfortably and look at the cross on the screen. The measurement will take five minutes.” We did *not* mention that eye blinks were being recorded.

### Cardiovascular measures

Systolic blood pressure change after clonidine administration was used as a proxy for central alpha-2 stimulation (Logemann et al. 2014), under the assumption that the effects of clonidine on cortical systems and the brainstem cardiovascular center are correlated. This is reasonable as the anti-hypertensive properties are probably at least partly mediated by stimulating alpha-2 receptors. Blood pressure and heart rate were assessed by an automatic blood pressure monitor (Microlife BP A6 PC) in a double-blind fashion.

### Motoric measures

The potential occurrence of hyperkinesia or akathisia (Kapur et al. 2000) after haloperidol administration was monitored with an accelerometer (Actigraph, GT3X+, Actigraph, LLC, Pensacola, FL, USA) placed around the right ankle of the participant. Motor activity along three axes (*x*, *y*, *z*) was stored with a sampling rate of 100 Hz. The increase in motor activity following haloperidol treatment was used as a proxy for central responsivity to haloperidol (Logemann et al. 2017).

### Subjective measures

The methods and analyses of the subjective effects data can be found in the supplementary materials, section 3.

### Procedure

During an initial screening session, the informed consent form was signed and in/exclusion criteria were checked. Figure 1B presents an overview of the procedure of each of the three main sessions. The procedure and room and lighting conditions were the same during all sessions. A cross-over design was used, and during each of the three sessions, participants either received 2 mg haloperidol, 0.150 mg clonidine, or placebo. Sessions were separated by at least one week. Participants and researchers who carried out the experiments and who performed the analyses were blinded to the drug allocation. Subjects were randomly assigned to a drug order, which was counterbalanced across subjects. Randomization of drug order was performed using Excel by a researcher who was not involved in the study.

### Data reduction and analysis

#### Cued Go/NoGo task—behavioral data

Mean reaction times (RTs) for valid responses to the target (i.e., single responses within the time window 150–1500 ms after target onset), reaction time variability (SDRT), the percentage correct responses, and the percentage omissions were calculated for each condition and each subject. Pre-drug behavioral data of one subject were not stored due to a technical issue. Behavioral data of this subject were therefore not analyzed.

#### Spontaneous movements

Ten minutes of data were analyzed for the pre- and post-drug condition, starting from 10 min after cued Go/NoGo task initiation. For each axis (i.e., the *x*, *y*, *z* direction), data were integrated into 10 s epochs and subsequently averaged across the three axes.

#### EBR and ERP data

Eye blink and ERP data were analyzed using Brainvision Analyzer 2.0 (Brain Products GmbH). Data were re-sampled to 256 Hz. Bipolar eye blink signals were obtained by subtracting the lower and upper channels, respectively. EEG data were re-referenced to the averaged mastoids. A 0.5–30-Hz band pass filter (24 dB/oct) and an additional 50 Hz notch filter were applied. The number of eye blinks were subsequently counted using the Gratton and Coles algorithm (Gratton et al. 1983) and transformed to EBR per minute.

ERP data were epoched into windows from − 100 to 1000 ms surrounding cue and no-cue onset (no-cues were letter stimuli not followed by a target and not preceded by a cue). Epochs with incorrect, pre-mature (< 150 ms) or late responses to the target (> 1500 ms), or with omissions or commission errors were excluded. For each subject, extreme artifacts in the EOG channels and the target midline electrodes^1^ were removed automatically without removing normal eye blink activity. This was done in order to improve the subsequent ocular artifact correction step, which used the Gratton and Coles algorithm (Gratton et al. 1983). Two subjects exhibited a very low number of eye blinks, which led to an incorrect estimation of vertical EOG transfer coefficients. Therefore, for these two subjects, the Gratton and Coles method was used to correct only horizontal EOG artifacts and to remove segments with vertical EOG artifacts.^2^ This was done for all task and drug conditions. Furthermore, for six subjects, EOG correction was inadequate for the posterior electrodes (Pz, POz, and/or Oz), as evidenced by clearly deviant EOG transfer coefficients for these electrodes. For these subjects, we used the EEG signal of the Pz, POz, and Oz leads without EOG correction for all task and drug conditions (the signals of the other electrodes were corrected for ocular artifacts).

Data were baseline corrected using the 100-ms period before cue onset. Segments with activity lower than 0.5 μV over a 100-ms period or with an absolute difference between values exceeding 100 μV were automatically removed. Average ERPs were computed for each condition, and ERP activity time-locked to no-cues was subtracted from cue-locked ERP activity for each condition. This was done to isolate blocked condition–dependent brain activity specifically associated with the cue. Finally, grand averages of the cue-minus-no-cue ERPs were computed for each condition.

Data of two subjects were discarded because of more than 50% choice errors and/or omissions for one or more conditions. Data of one subject were discarded because of inadequate ocular artifact correction, which was likely due to atypical eye movements. The final number of subjects included in the ERP analyses amounted to 23.

### Statistical analyses

#### Cued Go/NoGo task—behavioral data

Repeated measure MANOVAs (GLM, SPSS version 22) were run for RT, RT variability, the percentage correct responses, and the percentage omissions, with reward value (no reward, reward), target probability (50%, 98%), drug (clonidine, haloperidol, placebo), and time (pre-, post-drug) as within-subject variables. For each performance variable, we conducted an additional analysis including as between-subjects factors EBR-based and spontaneous movement-based median splits (for haloperidol versus placebo), and systolic blood pressure–based median split (for clonidine versus placebo).

For each subject and variable, linear and quadratic polynomial trend scores were computed for the time × drug × reward and time × drug × probability interactions. The distributions of these polynomial trend scores were tested for deviation from normality using Shapiro-Wilk’s tests. Non-parametric Wilcoxon signed rank (WSR) tests were conducted in cases where the linear and/or quadratic polynomial trend scores were not normally distributed. These non-parametric tests examined the change in the reward (or probability) contrast from the pre- to post-drug time point for the drug conditions versus the placebo condition. With respect to general drug effects (i.e., effects of drugs independent from task condition) and task condition main effects, results of WSR tests are reported for contrasts with a non-normal distribution.

Alpha was set at 0.05. The between-subjects factor drug order was initially included in all MANOVAs in order to reduce the variance induced by this factor for the tests of interest. If a given effect of interest did not depend on order, the order factor was removed from the model, so as to increase the dfs for the effect of interest (Kenemans et al. 1999).

#### Cued Go/NoGo task-ERP data—selection of time windows and electrodes

We originally started by selecting electrodes and time windows for statistical testing that had the strongest effects in our prior study (a priori selection method (Schutte et al. 2019)). This yielded 199–280 ms at FPz (reward-related positivity), 363–526 ms at CPz (reward P300), and 445–485 ms at FCz (probability-related positivity). These intervals are also largely consistent with data of and intervals used by prior studies (Flores et al. 2015; Holroyd et al. 2011). However, based on a recommendation during a previous review process, we alternatively implemented the “collapsed localizer” approach (CLA; Luck and Gaspelin 2017). The latter method involves testing where/when task effects (i.e., the main effect of reward and probability) are strongest across all drug conditions and using these electrodes/time windows to subsequently test the effects of the drugs. The a priori selection method may be less sensitive, because the spatial and temporal distributions of the task effects may be slightly different for different experiments. Although on the one hand, the CLA is likely to identify spatial-temporal segments based on drug conditions with large reward/probability effects (and probably larger than in other drug conditions), on the other hand, this may also yield insensitivity: Because task effects are tested across drugs, task effects may be missed if drugs have opposite effects on the sign of the reward and probability ERP amplitudes. For example, one would not find a main effect of probability across drugs if ERP amplitudes are larger for the high compared with low probability condition under placebo and haloperidol, and if this effect is reversed under clonidine (i.e., low probability > high probability). To foreshadow our results, this is exactly what happened. We could not identify electrode locations and time windows with a main effect of probability using the CLA. Therefore for the PRP, we chose the target electrode and time window with the strongest effect in our prior study (i.e., 445–485 ms at FCz). With respect to the RRP and reward P300, the CLA yielded 240–280 ms at FPz for the RRP, and 281-526 ms and the average of the electrodes CPz, Pz, POz, and Oz for the reward P300.

Effects of the drugs on the three ERPs were tested using the drug × reward value × probability MANOVAs. We noticed, however, that the results obtained with the two methods were slightly different, even for the PRP for which the same time window and electrode was used for testing. The latter was due to a difference in the artifact rejection procedure between both methods leading to a different selection of segments (see also footnote 1).

There is no specific reason to believe that for this study, one of these methods is superior to the other. We, therefore, chose to average the ERPs obtained with the two methods, under the assumption that the ERPs obtained with the a priori method reflect the same underlying processes as the ERPs obtained with the CLA. Averaging between these two methods suppresses the noise associated with each of these methods, while commonality between the methods is kept.

The ERP results section presents the results of ERP data averaged across selection methods. Supplementary materials, section 7, presents the ERP results derived from the two methods separately. Effects of the drugs on the three ERPs were tested using the drug × reward value × probability MANOVAs. Additional median split analyses were run to take individual variance in the effect of the administered dose into account. A proxy for individual variance in the effect of haloperidol on the reward P300 and RRP was based on spontaneous movement increase after haloperidol compared with placebo. A proxy for individual variance in the effect of clonidine on the PRP was based on the decrease in systolic blood pressure after clonidine compared with placebo. Another median split analysis addressed individual variance in baseline DA, for which EBR during the placebo condition was used as a proxy. For all median split analyses, we only compared the drug of interest with placebo.

Alpha was set at 0.05. As done for the behavioral analyses (“Cued Go/NoGo task—behavioral data” section), the between-subjects factor drug order was initially included and removed from the model if a given effect of interest did not depend on order. For each subject and ERP component, linear and quadratic polynomial trend scores were computed for the drug × reward and drug × probability interactions. The distributions of these polynomial trend scores were tested for deviation from normality using Shapiro-Wilk’s tests. For the ERP data, none of these distributions deviated significantly from normality.

#### Drug effects on peripheral measures

Additional analyses were run to test the group level effects of the drugs on EBR, cardiovascular data, and spontaneous movements. These results can be found in section 4 of the supplementary materials.

## Results

### Cued Go/NoGo task—behavioral results

For none of the four performance measures (RT, RT variability, % correct, % omissions), there were significant interactions among the effects of drug, reward value, and probability, respectively (all *p* > .079). With respect to main effects of reward value and probability (details can be found in the supplementary materials, section 5), subjects responded faster and were more accurate in the reward compared with the no reward condition. RTs were also less variable and the percentage omissions were lower for the reward compared with the no reward condition. RTs were also shorter and less variable in the high compared with the low probability condition.

**Fig. 2:**
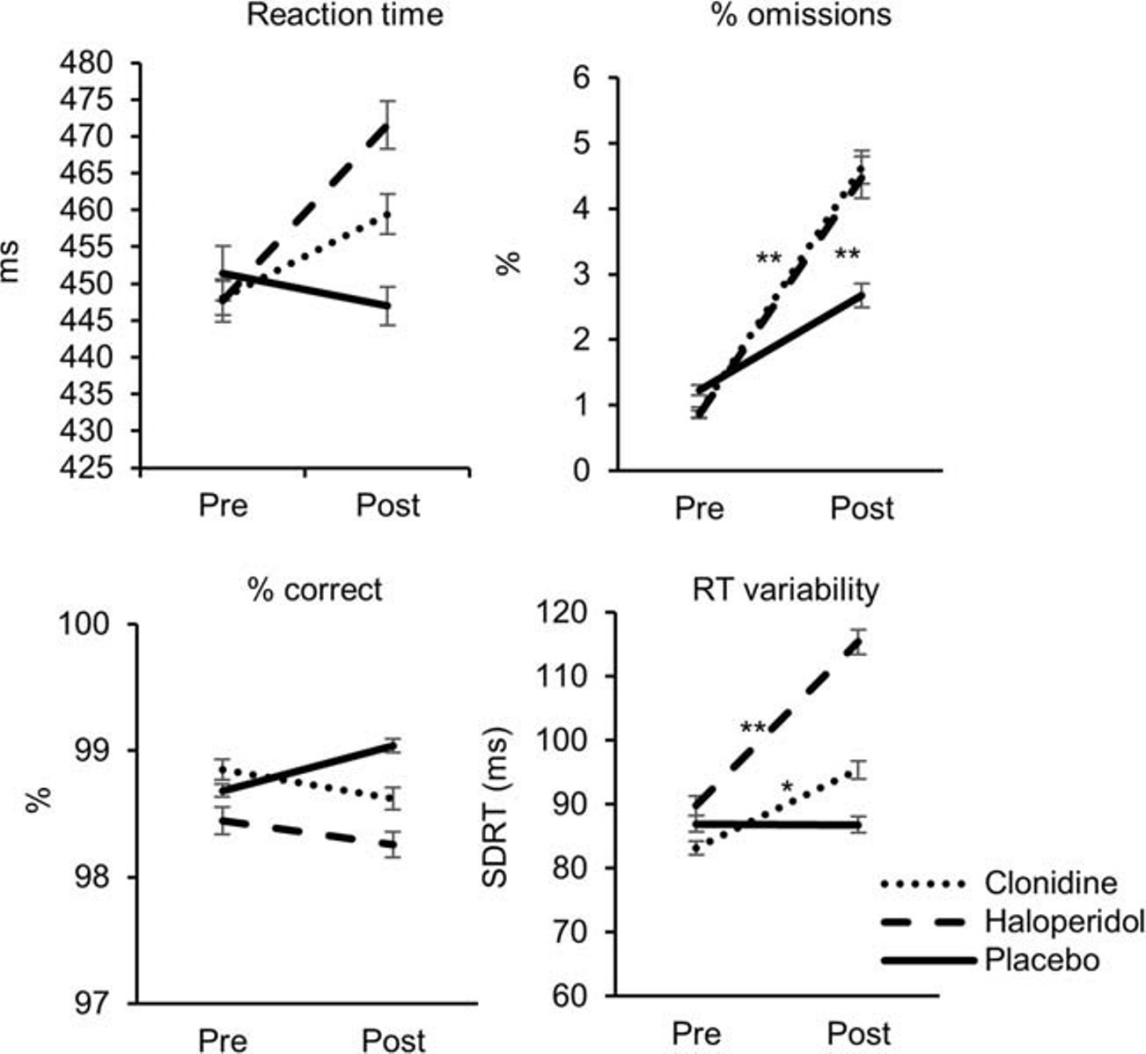
Effects of the medication on behavioral parameters. The figure presents the effects of drug administration on reaction times, the percentage omissions, the percentage correct responses, and the reaction time variability during the cued Go/NoGo task. Pre on the *x*-axis represents the pre-medication measurement; post represents the post-medication measurement. Error bars represent 1 standard error. Stars represent significant differences between the pre- and post-drug measurement; *p < .05, **p < .01

As to drug effects (Fig. 2), the percentage omissions across all task blocks was significantly increased after clonidine, *Z* = − 3.3, *p* = .001, rank-biserial *r* (rrb) = 0.82, and haloperidol administration, *Z* = − 3.4, *p* = .001, rrb = 0.89, compared with the pre-treatment time point (time × drug: *F*(2,20) = 5.2, *p* = .016, η_p_^2^ = 0.34). The increase from pre- to post-measurement was significantly stronger for haloperidol and clonidine compared with placebo, *t*(21) = 2.3, *p* = .032, Z = − 2.5, *p* = .014, respectively. A significant time × drug interaction was found for RT variability, *F*(2,20) = 3.9, *p* = .037, η_p_^2^ = 0.28, indicating that RT variability was increased following clonidine, *F*(1,21) = 8.0, *p* = .010, η_p_^2^ = 0.28 and haloperidol treatment, *F*(1,21) = 14.8, *p* = .001, η_p_^2^ = 0.41. No such increase was observed following placebo (*p* = .99). The increase in RT variability from pre- to post-treatment measurement was significantly stronger for both the clonidine and haloperidol condition compared with placebo (*p* values ≤ .042).

No significant interactions between time × reward × drug × EBR and time × reward × drug × spontaneous movement for haloperidol versus placebo were observed for any performance variable. The systolic blood pressure–based median split for clonidine versus placebo revealed a significant interaction between time × drug × probability × systolic blood pressure change for the percentage omissions, *F*(1,10) = 7.5, *p* = .021, η_p_^2^ = 0.43. There was only a significant interaction between time × drug × probability in subjects with a low clonidine-induced blood pressure change, *p* = .023. Follow-up tests for the low blood pressure change group did not reveal a significant time × probability interaction for either the clonidine or the placebo condition, *p* values ≤ .748.

### Cued Go/NoGo task—ERPs

#### Drug effects on the RRP

**Fig. 3:**
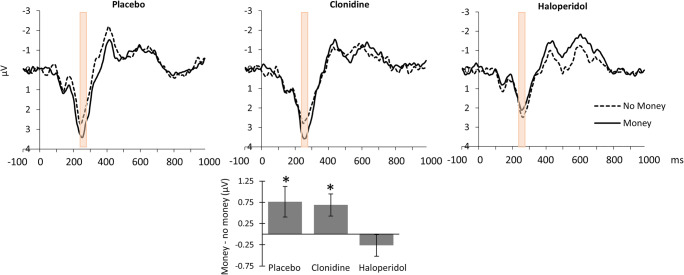
The reward-related positivity displayed for each drug condition. Top row: There was a significant drug × reward interaction for the RRP at electrode Fpz (*p* = .012). The RRP was significantly present for the placebo and clonidine condition (*p* = .02, and *p* = .023, respectively) but not for the haloperidol condition (*p* = .34). The data shown have been averaged across a priori and collapsed localizer (CLA) selection methods. Bottom row: The bar graph displays the average reward-no reward difference averaged across electrode FPz 199–280 ms (a priori method) and electrode Fpz 240–280 ms (CLA). Error bars represent ± 1 standard error. * *p* < .05

The RRP was significantly present across drug conditions (main effect of reward), *F*(1,17) = 5.6, *p* = .031, η_p_^2^ = 0.25. There was also a significant drug × reward interaction, *F*(2,16) = 6, *p* = .012, η_p_^2^ = 0.43. Follow-up tests showed that the RRP was significantly and specifically reduced by haloperidol, as evidenced by significant main effects of reward for both clonidine, *F*(1,17) = 6.2, *p* = .023, η_p_^2^ = 0.27 and placebo, *F*(1,17) = 6.6, *p* = .020, η_p_^2^ = 0.28, and absence of the reward effect for haloperidol, *p* = .335 (Fig. 3).

**Fig. 4:**
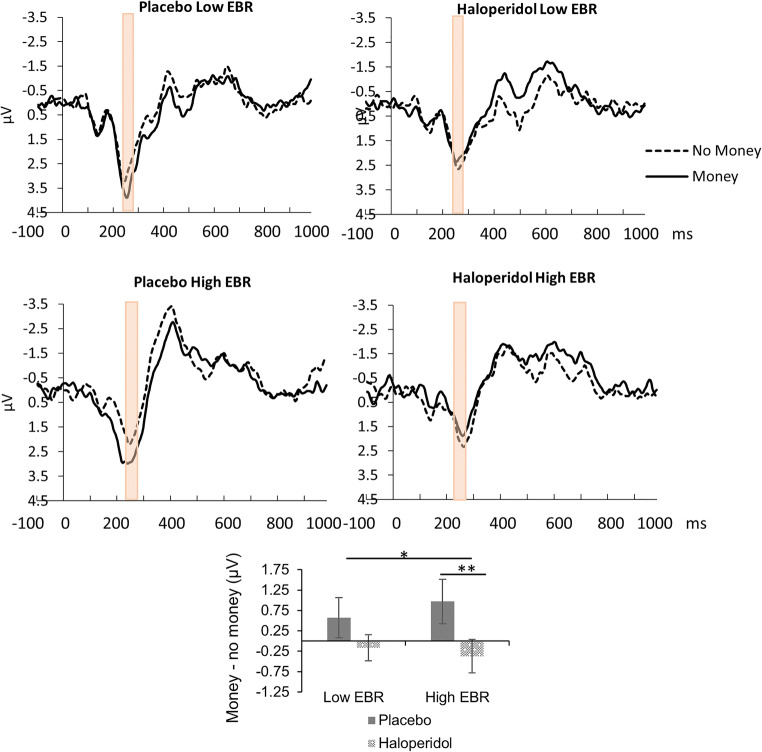
Drug effects on the reward-related positivity for subjects with high and low baseline dopamine activity. The attenuation of the reward-related positivity by haloperidol (compared with placebo) was more pronounced in subjects with high baseline dopamine activity (bottom row, high EBR). The top row presents the RRP under placebo and haloperidol in subjects with low baseline dopamine activity. The bar graph displays the average reward-no reward difference averaged across selection method 1 (i.e., electrode FPz, 199–280 ms) and method 2 (i.e., electrode FPz, 240–280 ms) for subjects with high and low baseline dopamine separately. Error bars represent ± 1 standard error. ^*^p < .05, **p < .01

The median split analysis including the factor EBR revealed a significant dissociation between the effect of haloperidol and placebo on the RRP in subjects with high and low baseline dopamine activity (Fig. 4), *F*(1,11) = 7.3, *p* = .021, η_p_^2^ = 0.4 (drug × reward × EBR group). Haloperidol significantly attenuated the RRP relative to placebo in subjects with high baseline dopamine activity, *F*(1,5) = 26.4, *p* = .004, η_p_^2^ = 0.84 (drug × reward); reward > no reward placebo, *p* = .001, η_p_^2^ = 0.9; reward > no reward haloperidol, *p* = .285. There was no significant difference between haloperidol and placebo with respect to the RRP amplitude in subjects with low baseline dopamine activity, *F*(1,6) = 1.2, *p* = .373 (drug × reward).

There was no such dissociation between drug responders and non-responders (based on movement increase following haloperidol), *p* = .446.

#### Drug effects on the reward P300

The reward P300 was significantly present across drug conditions when testing on the group level, main effect of reward, *F*(1,22) = 6.7, *p* = .017, η_p_^2^ = 0.23. There was no significant main effect of drug and also no interaction with drug when testing across all subjects. Fig. S6.1 displays the reward P300 for all drug conditions.

**Fig. 5:**
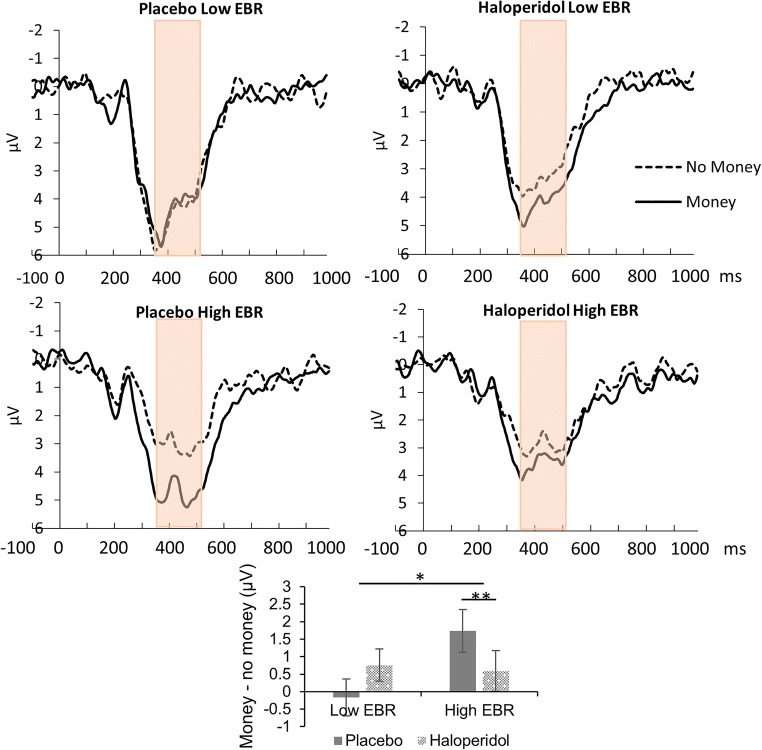
Drug effects on the reward P300 for subjects with high and low baseline dopamine activity. The effect of reward on the P300 was significantly attenuated under haloperidol compared with placebo, but only in subjects with high baseline dopamine activity (bottom row, high EBR). There was no such effect for subjects with low baseline dopamine activity (top row, low EBR). The interaction between reward and EBR in the placebo condition was significant, *p* = .046. The data shown have been averaged across a priori and collapsed localizer (CLA) selection methods. The bar graph displays the average reward-no reward difference averaged across selection method 1 (i.e., electrode CPz, 363–526 ms) and method 2 (i.e., averaged signal of electrodes CPz-Pz-POz-Oz, 281–526 ms), for subjects with high and low baseline dopamine separately. Error bars represent ± 1 standard error. ^*^p < .05, **p < .01

However, as shown in Fig. 5, the median split analysis revealed a dissociation between the effect of haloperidol and placebo on the reward P300 in subjects with high and low baseline dopamine activity, *F*(1,11) = 7, *p* = .025, η_p_^2^ = 0.38 (drug × reward × EBR group). Haloperidol significantly attenuated the reward P300 relative to placebo in subjects with high baseline dopamine activity, *F*(1,5) = 18, *p* = .008, η_p_^2^ = 0.78 (drug × reward), reward > no reward placebo, *p* = .011, η_p_^2^ = 0.76; reward > no reward haloperidol, *p* = .394. There was no significant difference between haloperidol and placebo with respect to the reward P300 amplitude in subjects with low baseline dopamine activity, *F*(1,6) = 1.2, *p* = .315 (drug × reward).

There was no such dissociation between drug responders and non-responders (based on movement increase following haloperidol), *p* = .831.

#### Drug effects on the probability ERP

**Fig. 6:**
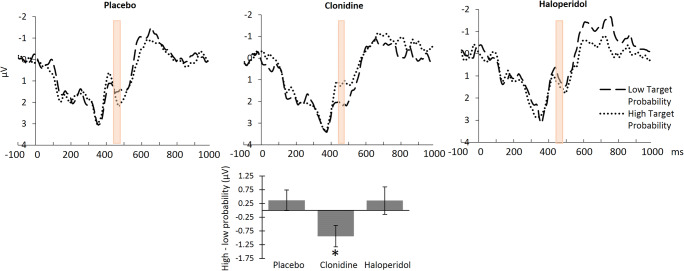
Drug effects on the probability-related positivity. Top row: A significant drug × target probability interaction was observed within the target time window of the PRP (displayed in pink) at electrode FCz (*p* = .017). Bottom row: ERP activity was significantly decreased for the high compared with low target probability blocks under clonidine. This pattern was reversed (but not significantly different) for the other two drug conditions. The data shown have been averaged across a priori and collapsed localizer (CLA) selection methods. The bar graph displays the average high-low target probability difference for electrode FCz between 445 and 485 ms. Error bars represent ± 1 standard error. *p < .05

There was no main effect of target probability on the cue-elicited ERP in the expected time window and sensor space. There was however a significant interaction between probability and drug (Fig. 6), *F*(2,21) = 5, *p* = .017, η_p_^2^ = 0.32. The probability effect was not significant (although in the expected direction, i.e., less positivity with low probability) under placebo (*p* = .346) or haloperidol (*p* = .486). It was significant, but in the opposite direction (less positivity with high probability) under clonidine *p* = .024, η_p_^2^ = 0.21.

There was no significant interaction between drug, probability, and systolic blood pressure increases, *p* = .114.

## Discussion

The current study supports the hypothesis that cortical mechanisms associated with the processing of reward value and of probability are directly related to the dopamine and noradrenaline neurotransmitter systems, respectively.

Cortical mechanisms related to the processing of prospective reward value reflected by the reward P300 and RRP were significantly reduced by haloperidol as expected. The reduction of the RRP as elicited by reward cues is consistent with prior studies showing that DA antagonists reduce the amplitude of the ERN (De Bruijn et al. 2006; Forster et al. 2017; Zirnheld et al. 2004). The ERN is an ERP component that is more negative following erroneous responding relative to correct responding and is possibly functionally related to the RRP.

The reduction of the RRP by haloperidol was most pronounced for participants with a high proxy for baseline DA levels (EBR). Furthermore, the attenuation of the reward P300 by haloperidol was only significant for participants with high EBR. The use of EBR as an indirect measure of tonic (baseline) striatal DA activity is well-established (Jongkees and Colzato 2016). The link between EBR and striatal DA activity is supported for example by evidence from genetic studies showing a relationship between EBR and DA gene polymorphisms (Dreisbach et al. 2005), and by effects of DA drugs on EBR (Blin et al. 1990; Karson 1983). In a recent study by Dang et al. (2017), no correlation was observed between dopamine D2 receptor availability and EBR. Another study (Sescousse et al. 2018) found a tentative negative correlation between 18F DOPA binding capacity and baseline EBR. In the supplementary materials, section 9, we argue that the findings by Dang et al. (2017) and Sescousse et al. (2018) are not necessarily inconsistent with the hypothesis of EBR being a proxy of dopamine function.

The stronger reduction of the RRP and reward P300 by haloperidol in subjects with high EBR is consistent with the notion that the effects of DA medication may differ between individuals depending on baseline DA level (Cavanagh et al. 2014; Cools et al. 2009; Frank and O'reilly 2006; Martins et al. 2017). This finding is particularly interesting in light of the relatively greater efficacy of DA antagonists in patients with high baseline DA levels such as seen in patients with psychotic symptoms (Abi-Dargham et al. 2000). Individual differences in the effect of DA drugs depending on baseline DA may explain the mixed findings in the literature (Frank and O'reilly 2006) with respect to the direction of the DA effects. It has been suggested that single low doses of haloperidol including 2 mg exclusively stimulate pre-synaptic D2 receptors and therefore result in an acute increase of DA transmission (Frank and O'reilly 2006). However, the current data as well as numerous other experimental results (e.g., reduced approach (Pessiglione et al. 2006), reduced inhibitory control (Logemann et al. 2017), and increased prolactine levels (Frank and O'reilly 2006) after haloperidol 1 and 2 mg, respectively) indicate that this may not hold so much in general. The pre-/post-synaptic effect balance may be a matter of differences in baseline DA activity. For example, in high baseline DA subjects, there may be relatively more post-synaptic binding because of pre-synaptic D2 receptor occupancy by endogenous DA. The current results suggest, however, that there was more room for reduction of the P300 and RRP amplitude by haloperidol in the high baseline DA group because of a relatively increased reward P300 and RRP under placebo in the high compared with the low baseline DA group. In any case, the observed relationship between DA drug effects and a proxy for endogenous DA demonstrates that it is useful to take endogenous DA into account in future studies.

The ERP component associated with the processing of the probability of a prospective (rewarded) target (PRP) was specifically related to the NA manipulation as expected. Specifically, the PRP was larger (although not significantly) in the high versus the low probability condition under placebo and haloperidol, and this effect was reversed (i.e., significantly larger in low versus high probability condition) under clonidine. This is consistent with our hypothesis of a PRP being reduced specifically under clonidine. However, this effect pattern also prompts the assumption of an additional influence to account for the absence of a PRP in especially the placebo condition (which was pronounced in our previous study, Schutte et al. 2019). One possibility is the difference in gender ratios between the two studies (all males in the current study versus a mixed sample in our previous study). Another option refers to context dependency of clonidine effects (Brown et al. 2015; de Rover et al. 2015). Differences in context between our previous and our current study may concern the current repetition of the various conditions across three sessions (as opposed to just one in the previous study).

With respect to behavioral performance, both drugs reduced detection rates and increased variability in response speed in a non-specific manner, indicative of reduced attention (Logemann et al. 2017). This pattern of behavior may alternatively be explained in terms of increased fatigue, as both drugs induced increased subjective feelings of tiredness compared with placebo. The drugs did, however, not significantly slow reaction times, which speaks against this interpretation in terms of increased fatigue. Furthermore, the attenuation of the reward P300 following haloperidol is not easily explained in terms of decreased attention or increased fatigue as this effect was specific for a group of subjects with high baseline dopamine levels. This suggests that it reflects direct effects of the drug on systems underlying this ERP rather than reflecting a general effect on attention/alertness during task performance.

A limitation of the current study concerns the repeated sessions, which may in its own right have altered the pattern of the PRP. Also, and in contrast to our previous study, the exclusive focus on male participants may be seen as a limitation. A future replication should perhaps employ a between-subjects design and include female participants, taking into account natural hormonal fluctuations.

In conclusion, the current study demonstrates that cortical processes related to the anticipation of reward value and probability relate to the dopaminergic and noradrenergic system, respectively. These results may have implications for the pharmacological treatment of patients displaying problems with reward processing and decision-making, such as patients with depression, schizophrenia, and ADHD. Treatments for these disorders often target the NA or DA system (El Mansari et al. 2010). Future studies could investigate whether reward and probability ERPs predict the effectiveness of DA and NA treatment in these patients. For example, patients with an attenuated reward P300 may benefit more from compounds targeting the DA system. Furthermore, the current study demonstrates that it is useful to take baseline DA functioning into account when investigating the effects of DA drugs.

## Electronic supplementary material

ESM 1 (DOCX 3290 kb)

## Footnotes

1 As will be explained in the paragraph “Cued Go/NoGo task - ERP data –selection of time windows and electrodes,” we used two methods to select target time windows and electrodes for statistical testing. For the selection method based on the results of our prior study, segments were removed in case of extreme artifacts in only the EOG channels or the channels of a priori interest, i.e., FPz, FCz, or CPz. For the collapsed localizer approach, segments were removed in case of extreme artifacts in the EOG channels or any of the midline electrodes (from FPz to Oz).

2 This was done for the collapsed localizer approach. Eye blink correction was adequate for the channels of a priori interest (FPz, FCz, CPz) in these two subjects.
