# Supplementary material for: Dopaminergic and noradrenergic manipulation of anticipatory reward and probability event-related potentials
Source: Psychopharmacology (Berl). 2020 Apr 20;237(7):2019–30. doi: 10.1007/s00213-020-05515-x (PMC7306042; doi:10.1007/s00213-020-05515-x)
Supplement: Supplementary file 1 — (DOCX 3290 kb) [file 213_2020_5515_MOESM1_ESM.docx]

**Supplementary materials**

**1. Sample size justification**We aimed at a final sample of 28 subjects plus two extra subjects (for drug order balancing purposes). This sample size (n=28) was based on a prior study in which the effects of haloperidol 2 mg were compared to placebo on stop signal reaction times (SSRTs) (Logemann et al., 2017). In this study haloperidol was found to significantly increase SSRTs. Note that, like SSRT (derived from successful-stop rate and go RT), our variables of interest are derived from a combination of underlying variables, e.g., the RRP consists of the difference between the ERP in the reward and that in the no-reward condition. Due to time/ end of contract restriction we were forced to at least temporarily suspend inclusion at n=26 and performed interim analyses. This resulted in 3 further exclusions. Given the results with respect to the hypothesized drug x task factor effects we decided that it was no longer necessary to include (and burden) further participants.

**2. Cued Go/NoGo task and randomisation**

During this task the letters A, C, D, E, F, G, H, J, L, X and Y (in black, font size 79) were presented in the center of a grey colored screen and between two vertical bars (height: 1.03º, width: 0.05º). Letter stimuli were presented for 150 ms followed by inter-trial intervals with a random duration between 1400 and 1600 ms. Subjects had to press a left or right button when a target letter (letter X or Y) followed a cue letter (always letter A). Each of the target letters (X, Y) was associated with either the left or right button. This mapping was counterbalanced across participants. The response buttons were the “z” and “/” key of a qwerty keyboard. The letters of the keyboard were covered by a sheet and the target buttons were covered by a white sticker. Subjects were instructed to respond (or not) as fast and accurately as possible. The probability of target appearance after the cue (either 50 % or 98 %) and the amount of money that could be won for correct and fast responses (a total of either 0 Euros or 5 (or 2.5) Euros during the block) were orthogonally manipulated across four task blocks. This information was shown to participants at the beginning of each block. Participants could win 2.50 Euros during reward blocks of the pre-drug task version, because this task version was twice as short as the post-drug task version.

During the first session of the study and before the pre-drug cued Go/NoGo task version participants received a practice block consisting of 100 trials (letters). The practice block always consisted of the reward-98% target probability condition. During the second and third session participants received a shorter practice block consisting of 25 trials. The pre-drug cued Go/NoGo task version consisted of four blocks of 200 letter trials and the post-drug cued Go/NoGo task version consisted of four blocks of 400 letter trials.

One-minute rest breaks were provided between task blocks and halfway through each task block. Breaks within blocks were followed by a reminder of the target probability and reward availability of the current block.

In both the pre- and post- cued Go/NoGo task cues appeared with a frequency of 20 % during each block. Both the X and Y appeared with a frequency of 10%. In the 50% target probability blocks, half of the cues were followed by a target (50% X, 50% Y). The other half of the cues were not followed by a target (X or Y) and 50% of the X and Y stimuli were not preceded by a cue. Similarly, in the 98% target probability blocks, 98% of the cues were followed by a target (50% X, 50% Y). Two percent of the cues were not followed by a target and 2% of the targets (50% X, 50%Y) were not preceded by a cue. All other letters appeared with a frequency of 5%, except for letter H and C. These letters appeared with a frequency of 20% and 10%, respectively, in order to control for the frequency differences between cues, targets and other letters (Bekker, Kenemans, & Verbaten, 2004).

Letters were presented in a pseudo-random order, with the following restrictions: (1) the same letter was never repeated on the subsequent trial, (2) sequences of cues followed by targets (A-X, A-Y) and of cues not followed by targets were never followed by A, X, or Y.

Subjects were randomly allocated to one of the six drug orders. Each of the drug orders was used four times. Each subject of each drug order group was pseudo-randomly assigned to one of two task versions (which differed with respect to the hand-target mapping). Each task version was used twice within each drug order group. Furthermore, four task condition orders were created in which the order of reward (no reward or reward blocks first) and the order of probability (50% or 98% blocks first) were counterbalanced:

Order A: reward/50%, reward/98%, no reward/50%, no reward/98%

Order B: reward/98%, reward/50%, no reward/98%, no reward/50%

Order C: no reward/50%, no reward/98%, reward/50%, reward/98%

Order D: no reward/98%, no reward/50%, reward/98%, reward/50%

For each participant this order was kept the same for the pre- and post-version of the cued Go/NoGo task and for all three sessions. Task condition order balancing was maintained in such a way that the order of the reward blocks (the no reward blocks or the reward blocks first) was counterbalanced within each drug order group. For one of the drug order groups (hp-cld-pla) the order of the reward and no reward blocks ended up not perfectly counterbalanced (i.e., the order with reward-blocks-first occurred three instead of two times).

**3. Subjective measures**

*Methods*

Subjective effects of the drugs on mood were assessed by the Dutch short version of the Profile of Mood States (POMS) questionnaire (Wald & Mellenbergh, 1990). This self-report questionnaire consists of 32 statements describing feelings like “sad” and “tired” and comprises six subscales: depression, fatigue, tension, anger, vigor and total mood disturbance. Each item is rated on a scale ranging from “not” (score 0) to “very much” (score 4). The total mood disturbance score was computed by subtracting the total score from the vigor subscale from the total score of the other subscales.

Data from one subject were removed from the anger subscale, data from two subjects were removed from the fatigue subscale, and data from three subjects were removed from the total mood disturbance scale, because of incomplete responses.

MANOVAs (GLM, SPSS version 22) were run with drug and time (pre-, post-medication) as within-subjects variables. For each subject and subscale linear and quadratic polynomial trend scores were computed for the time x drug interactions. The distributions of these polynomial trend scores were tested for deviation from normality using Shapiro-Wilk’s tests. Non-parametric Wilcoxon signed rank (WSR) tests were conducted in cases where the linear and/or quadratic polynomial trend scores were not normally distributed. These non-parametric tests examined the change in mood scores from the pre to post drug time points for the drug conditions versus the placebo condition.

*Results*

Figure S3.1 displays the subjective mood scores over time for each drug condition. Only the levels of fatigue, vigor, and total mood disturbance were significantly affected by the drugs. Specifically, participants felt significantly more tired after administration of clonidine and haloperidol, but not after placebo, F(4,17) = 5.8, p = .004, η_p_^2^ = .58 (time x drug; Note that the distributions of the polynomial trend scores for this interaction were not normal. Results of non-parametric paired-samples WSR tests were therefore reported below), main effect of time haloperidol: p = .008, η_p_^2^ = .40, main effect of time clonidine: p < .001, η_p_^2^ =.64. The level of tiredness under clonidine and haloperidol was increased for the post1 compared to pre-drug time points (p’s ≤ .006). The increase was significantly stronger for clonidine compared to placebo and haloperidol (p’s < .001), and did not significantly differ between haloperidol and placebo (p = .225).

Mood disturbance scores increased significantly after drug administration for all drug conditions, but the effect was most pronounced for clonidine, which explains the time x drug interaction, F(4,16) = 5.4, p = .006, η_p_^2^ = .57 (note that the distributions of the polynomial trend scores for this interaction were not normal. Results of non-parametric paired-samples WSR tests were therefore reported below). The increase from the pre to post1 time point was significantly stronger for clonidine compared to placebo and haloperidol (p’s ≤ .017). There was no such difference between haloperidol and placebo (p = .79).


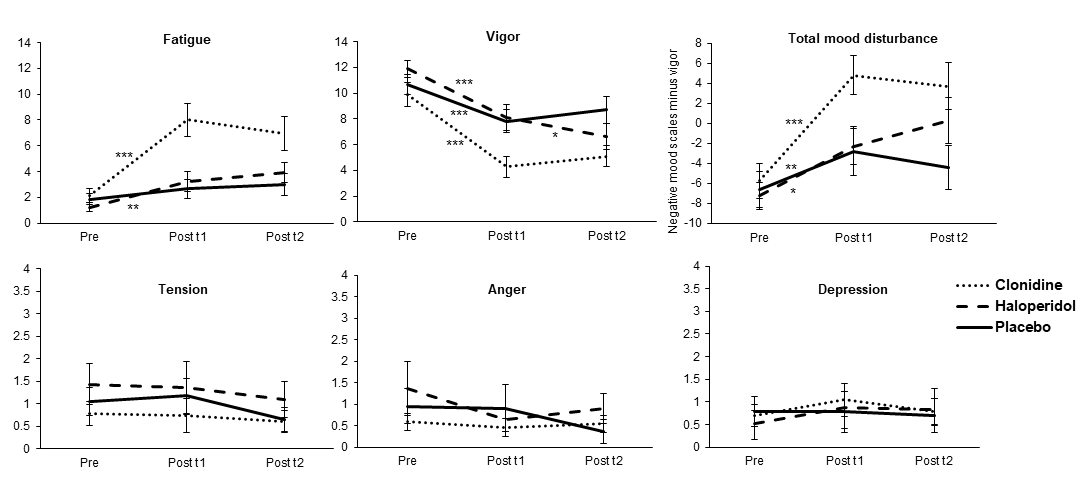
Subjects felt less vigorous after drug administration in all drug conditions. However, the drop in vigor was most pronounced for the clonidine condition explaining the time x drug interaction, F(4,19) = 5.1, p = .006, η_p_^2^ = .52. A stronger drop in vigor between the pre and post1 time point was observed for clonidine compared to placebo (p = .001), but there was no significant difference between haloperidol and, respectively, clonidine and placebo. The level of vigor stabilized from the post1 time point onwards under clonidine and placebo, whereas it dropped further under haloperidol (post1 – post2 time point under haloperidol: p = .021).

**Figure S3.1 Subjective mood scores.** Fatigue, vigor and total mood disturbance scores changed over time dependent on the drug condition. Error bars represent 1 standard error. Star symbols mark significant differences between two time points within a drug condition. * p < .05, ** p < .01, *** p < .001.

**4. Peripheral control measures***Spontaneous movements and eye blinks*
Additional MANOVAs were run to investigate whether the proxy for individual variance in the effect of haloperidol (spontaneous movements) and the proxy for individual variance in endogenous DA (eye blink rate, (EBR)) were affected by the drugs on the group-level. We expected spontaneous movements to increase after haloperidol treatment compared to placebo and clonidine. EBR was expected to decrease specifically after haloperidol treatment. Normality was tested as described in paragraph 2. The between-subjects factor drug order was initially included in the MANOVAs in order to reduce the variance induced by this factor. If a given effect of interest did not depend on order, the order factor was removed from the model, so as to increase the dfs for the effect of interest (Kenemans, Wieleman, Zeegers, & Verbaten, 1999). MANOVAs were run with drug and time (pre-, post-medication) as within-subjects variables.

Spontaneous movements were not significantly increased following haloperidol treatment (time x drug: F < 1, p = .477; Note that the distribution was non-normal. Non-parametric WSR tests comparing pre-post changes in spontaneous movements between the drug conditions and placebo did not reveal significance). The number of eye blinks during resting state was also not significantly different under haloperidol treatment compared to placebo and clonidine (F(2,21) = 1.3, p = .284). Figure S4.1 displays EBR and spontaneous motor activity during each drug condition.

As outlined above, spontaneous movements and EBR were not significantly affected by haloperidol when testing at the group-level. The effects of haloperidol on spontaneous movements were in the expected direction (post-treatment increase relative to placebo and clonidine). The non-significant effects may be explained by sizeable individual variance in e.g. pharmacokinetics, D2 receptor availability, and endogenous DA, possibly resulting in strong individual variance in the effect of haloperidol on these parameters. At least for EBR strong individual variance in the effects of DA drugs (DA agonist treatment) has been observed before (Cavanagh, Masters, Bath, & Frank, 2014). It was tested whether the expected drug-induced change in EBR (haloperidol vs placebo) was dependent on baseline EBR (i.e., placebo EBR), but there was no such dependency, p = .8 (drug*baseline EBR group).

*Cardiovascular data*Additional MANOVAs with drug and time (pre-, post-t1, post-t2 time point) as within-subjects variables were run for systolic and diastolic blood pressure and heart rate in order to test whether these parameters were affected by clonidine at the group-level. Time point T2 after drug administration was included in the test in order to examine whether the effects of clonidine on systolic blood pressure were evident until after the cued Go/Nogo task. We expected that clonidine would attenuate systolic and diastolic blood pressure. No effect was expected with respect to heart rate. Normality was tested as described in paragraph 3 (Subjective measures).

A significant time x drug interaction was found for systolic and diastolic blood pressure, F(4,19) = 17.7, p < .001, η_p_^2^ = .79; F(4,19) = 22.1, p < .001, η_p_^2^ = .82, respectively. Systolic blood pressure was significantly attenuated specifically following clonidine administration (Figure S4.1). None of the treatments significantly affected heart rate.

The effects of clonidine on cardiovascular variables were as expected. Figure S4.1 (right panel) shows that the attenuation of systolic blood pressure was evident until after the cued Go/NoGo task (time point T2). Systolic blood pressure remained stable between the time point three hours after drug administration (just before the cued Go/Nogo task) and time point T2 (after the task).

**
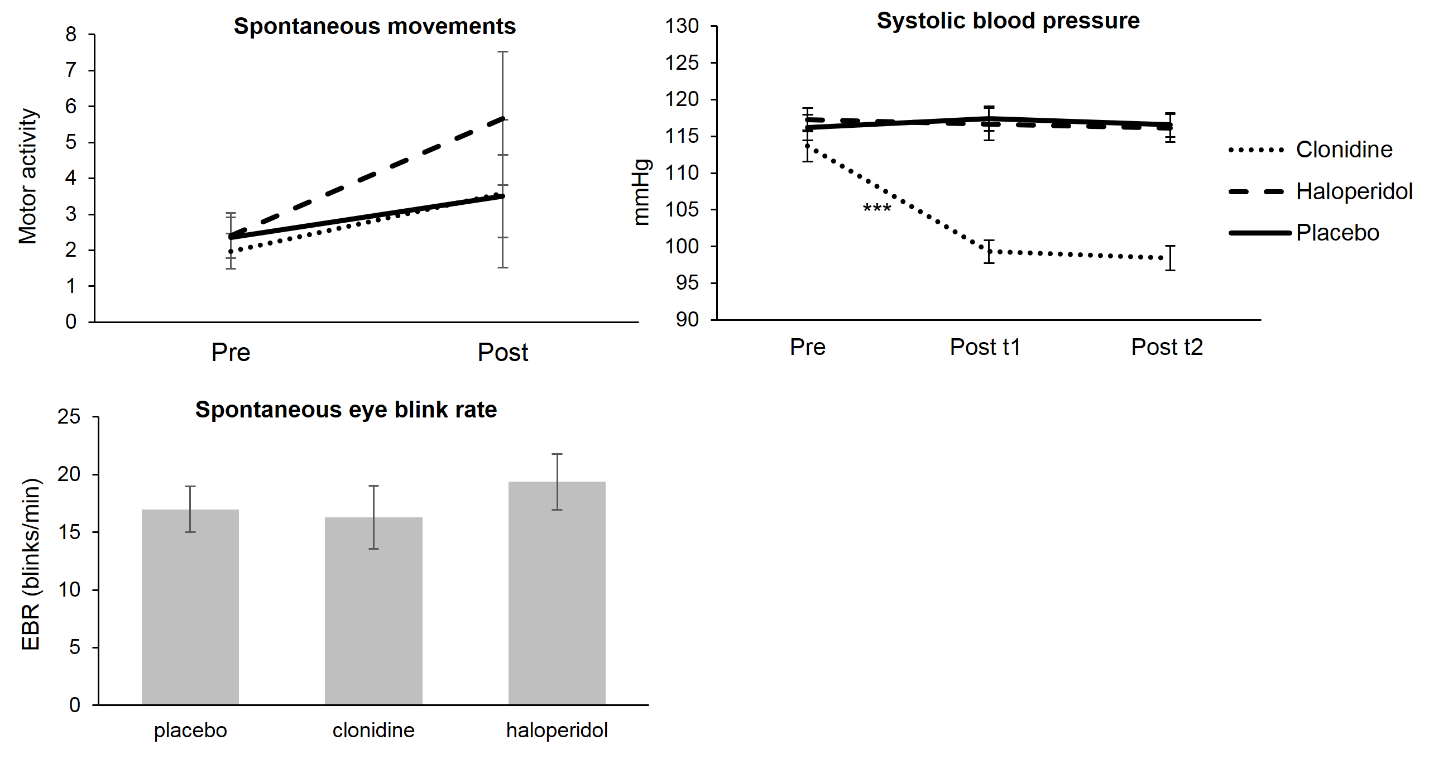
**

**Figure S4.1. Peripheral control measures.** Top left: spontaneous motor activity measured during the cued Go/NoGo task before (pre) and 3 hours after (post) drug administration. Data were collapsed across the x,y, and z movement direction. Top right: systolic blood pressure for each drug condition measured at 3 time points: before drug administration (Pre), at t = 2 h 47 min after drug administration (post t1), and at t = 4 h 22 min after drug administration (post t2). Bottom left: eye blink rate in blinks per minute for each drug condition. EBR was measured at t = 2 h 40 min after drug administration. Error bars represent 1 standard error. *** p < .001.

**5. The effects of reward and probability on behavioral parameters**With respect to main effects of reward value and probability (Figure S5.1), subjects responded faster and were more accurate in the reward compared to the no reward condition; F(1,21) = 30.7, p < .001, η_p_^2^ = .59; Z = -2.8, p = .005, rank-biserial r (rrb) = .72, respectively. RTs were also less variable and the percentage omissions was lower for the reward compared to the no reward condition, especially after treatment (time x reward interaction). Main effect of reward post-treatment: F(1,21) = 29.0, p < .001, η_p_^2^ = .58 (RT variability); Z = -3.0, p = .003, rrb = .74 (omissions). RTs were also shorter and less variable in the high compared to the low probability condition; Z = -4.1, p < .001, rrb = 1.0; F(1,21) = 9.2, p= .006, η_p_^2^ = .30, respectively.

**
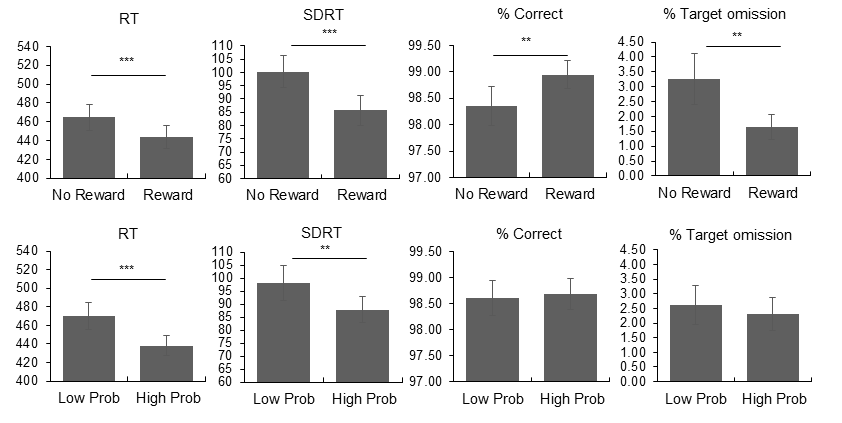
**

**Figure S5.1. Effects of reward and probability on behavioral parameters.** The figure presents the main effects of reward (top row) and target probability (bottom row) on reaction times, reaction time variability the percentage correct responses, and the percentage omissions during the cued Go/NoGo task. Data are averaged across all drugs and across the pre and post time points. Error bars represent 1 standard error. * p < .05, ** p < .01, *** p < .001.

**6. The reward P300 for each drug condition separately**

**
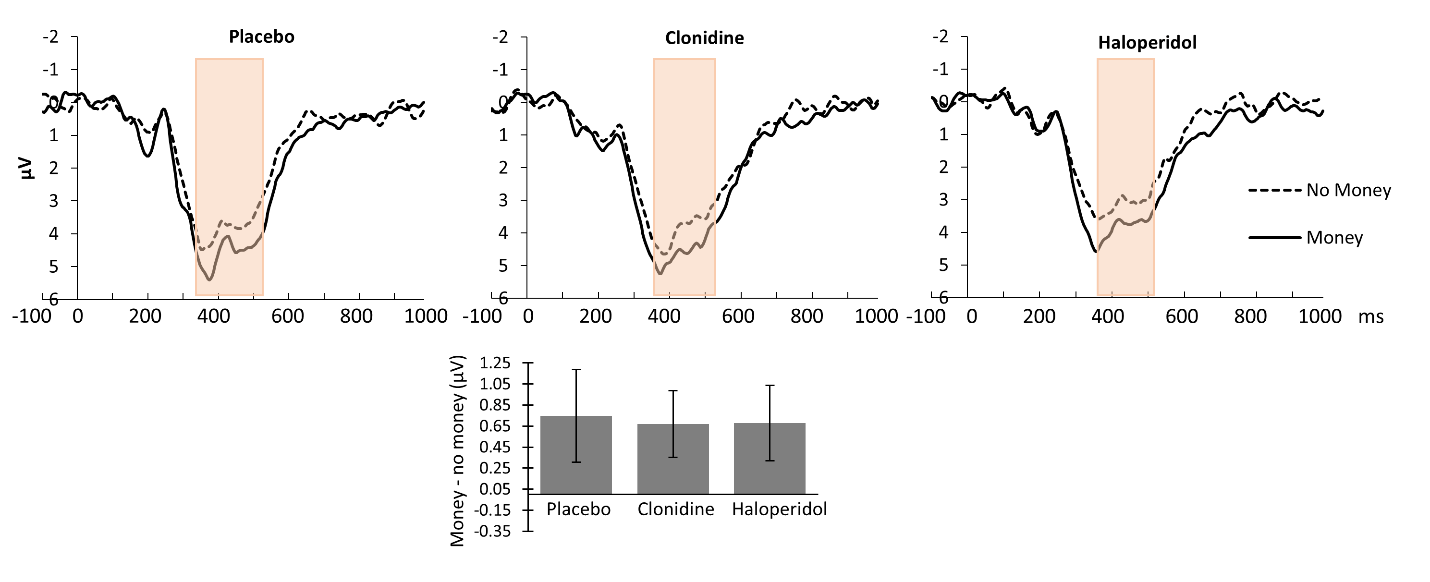
**

**Figure S6.1. Drug effects on the reward P300.** P300 activity was in general significantly increased for the money compared to the no money blocks. This reward effect was not affected by drug condition when tested across all subjects. The data shown have been averaged across a-priori and collapsed localizer (CLA) selection methods (see paragraph Cued Go/NoGo task - ERP data –selection of time windows and electrodes of the main paper). The bar graph displays the average reward-no reward difference averaged across selection method 1 (i.e., electrode CPz, 363-526 ms) and method 2 (i.e., averaged signal of electrodes CPz-Pz-POz-Oz, 281-526 ms). Error bars represent ± 1 standard error.

**7. Summary of the ERP results for the apriori and collapsed localizer approach separately**

**Apriori method**

**RRP (199-280 ms at Fpz)**The RRP was significantly present across drug conditions (main effect of reward), F(1,22) = 5.7, p =.026, η_p_^2^ = .21, but was not significantly different between drug conditions (drug*reward), F(2,21) = 1.6, p= .23. The drug*reward interaction was significantly dependent on EBR (drug*reward*EBR), F(1,11) = 14.6, p=.003, η_p_^2^ = .57. The drug*reward interaction was only significant in subjects with high EBR, p = .01, reward > no reward placebo: p = .001, reward > no reward haloperidol: p = .62. The drug*reward interaction was not significant for subjects with low EBR, p = .4.

**Reward P300 (363-526 ms at CPz)**

The reward P300 was not significantly different between drug conditions (drug*reward), F(2,16) < 1, p = .91. The drug*reward interaction was significantly dependent on EBR (drug*reward*EBR), F(1,11) = 8, p = .016, η_p_^2^ = .42. The drug*reward interaction was only significant in subjects with high EBR, p = .004, reward > no reward placebo: p = .009, reward > no reward haloperidol: p = .72. The drug*reward interaction was not significant for subjects with low EBR, p = .32.

**PRP (445-485 ms at FCz)**

There was a significant interaction between probability and drug, F(2,21) = 5.8, p=.01, η_p_^2^ = .36. The probability effect was not significant (although in the expected direction, i.e., less positivity with low probability) under placebo and haloperidol (p’s < .59). It was significant, but in the opposite direction (less positivity with high probability) under clonidine p = .008, η_p_^2^ =.28.

**Collapsed localizer approach**

**RRP (240-280 ms at Fpz)**

The RRP was significantly different between drug conditions (drug*reward), F(2,16) = 9.1, p = .002, η_p_^2^ = .53. The interaction with EBR did not reach significance (drug*reward*EBR), p= .28.

**Reward P300 (281-526 ms, average of CPz, Pz, POz, Oz)**

The reward P300 was significantly present across drugs, F(1,17) = 10, p = .006, but not significantly different between drug conditions (drug*reward), F(2,16) < 1, p = .97. The interaction between drug, reward, and EBR was marginally significant, p = .08.

**PRP (445-485 ms at FCz)**

There was a significant interaction between probability and drug, F(2,21) = 4, p=.03, η_p_^2^ = .28. The probability effect was not significant (although in the expected direction, i.e., less positivity with low probability) under placebo and haloperidol (p’s < .69). It was marginally significant, but in the opposite direction (less positivity with high probability) under clonidine p = .073, η_p_^2^ =.14.

**8. Spatial distribution of the RRP, reward P300, and PRP**

Figures 8.1-8.3 below display, respectively, the RRP, reward P300, and PRP averaged across drug conditions. Data are visualized for the midline electrodes and for different 40-ms time windows (as indicated). Preprocessing steps of the collapsed localizer approach (see main article) were used. Note that the drugs had opposite effects on the sign of the PRP, which explains the amplitude of approximately zero at FCz between 445-485 ms.


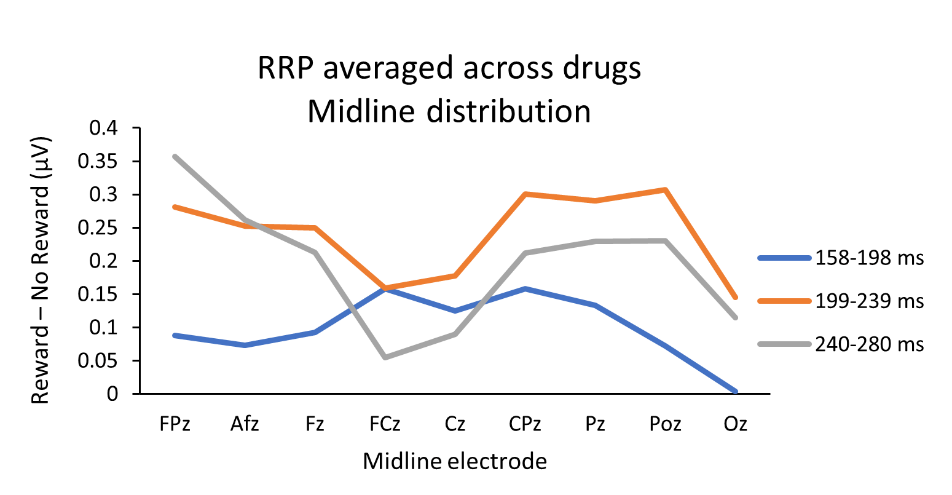


**Fig 8.1**

**
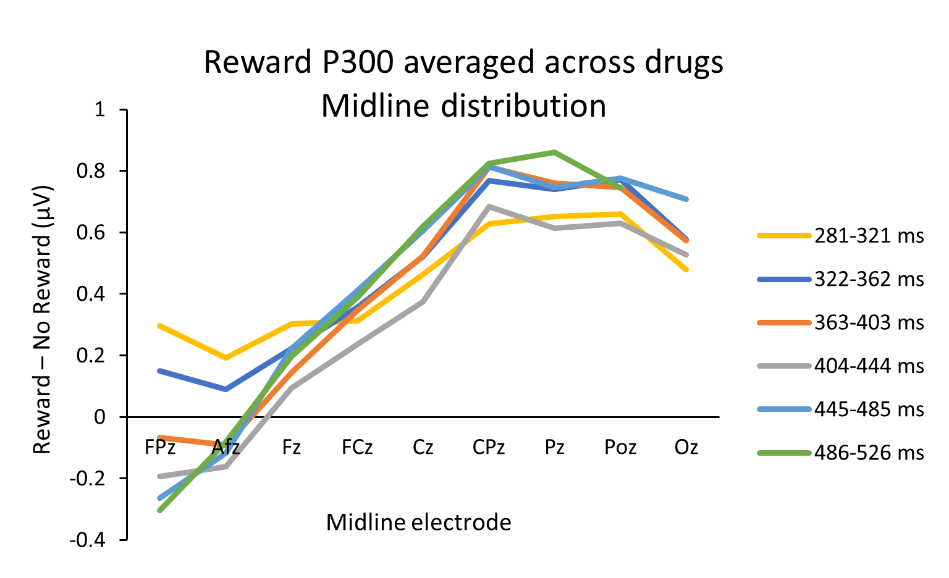
**

**Fig 8.2**

**
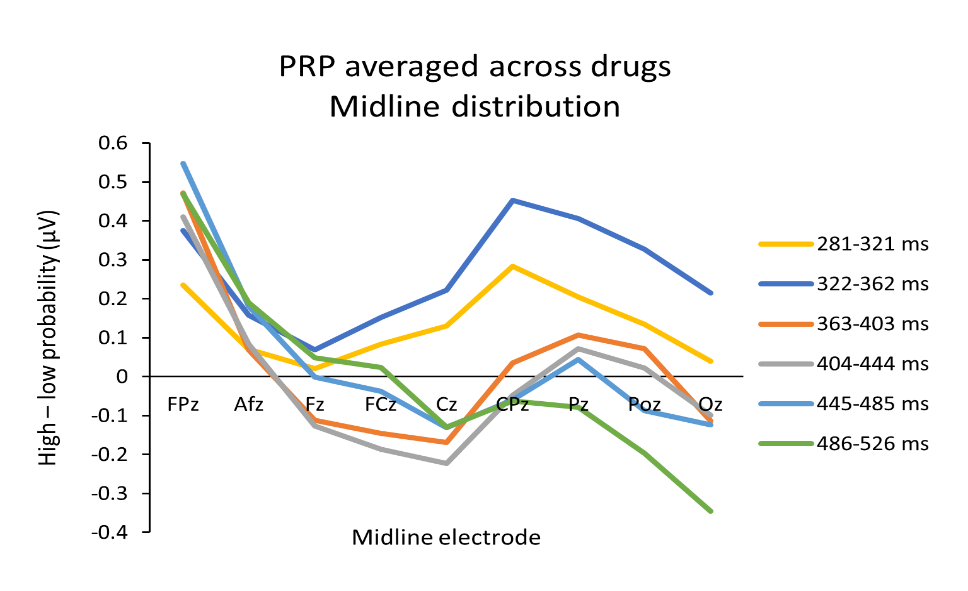
**

**Fig 8.3**

**9. EBR as a proxy of dopamine function**

In a recent study by Dang et al. (2017) no correlation was observed between EBR and dopamine D2 receptor availability. Furthermore, EBR was not modulated by dopamine agonism by bromocriptine in that study. These findings may seem at odds with the hypothesis of EBR as a proxy of dopamine function. However, we would like to present three arguments why the findings of Dang et al. (2017) are not necessarily inconsistent with the hypothesis of EBR as a proxy of dopamine function. First, the hypothesis of an EBR-dependent haloperidol effect is actually a constituted hypothesis: EBR reflects dopamine level AND dopamine level affects DA-antagonist response. If EBR indeed affects DA-antagonist response, that is consistent with both constituent hypotheses being true. If not, then either of the constituents, or both, may be false. Second, the Dang (2017) study finds no relation between baseline EBR and dopamine D2 receptor binding potential; but it is possible that, e.g., high-EBR individuals have elevated dopamine D2 receptor levels but also elevated DA levels, which could mask potentially elevated binding potential (to control for this something like DA depletion preceding the scan would be needed). Third, the lack of effect of bromocriptine on EBR is, although it is an agonist rather than an antagonist, consistent with the lack of a haloperidol effect on EBR as presently reported.

Another study (Sescousse et al., 2018) found a tentative negative correlation between 18F DOPA binding capacity and baseline EBR (with low EBR presumably reflecting low endogenous synaptic DA levels). This could be due to low synaptic DA levels being caused by low *endogenous* dopamine precursor (including DOPA) availability. Low precursor activity, in turn, may lead to higher binding potential for *exogenous* 18F DOPA with both the lat1/cd98 trans-membrane transporter (which transports precursors across the cell membrane) and the intracellular rate-limiting enzyme (decarboxylase) for metabolizing precursors (including DOPA to dopamine conversion; see Rakshi et al., 1999). This scenario is consistent with a positive relation between EBR and endogenous dopamine levels.

**References**

Bekker, E. M., Kenemans, J. L., & Verbaten, M. N. (2004). Electrophysiological correlates of attention, inhibition, sensitivity and bias in a continuous performance task. *Clinical Neurophysiology, 115*(9), 2001-2013.

Cavanagh, J. F., Masters, S. E., Bath, K., & Frank, M. J. (2014). Conflict acts as an implicit cost in reinforcement learning. *Nature Communications, 5*, 5394. doi:10.1038/ncomms6394 [doi]

Dang, L. C., Samanez-Larkin, G. R., Castrellon, J. J., Perkins, S. F., Cowan, R. L., Newhouse, P. A., & Zald, D. H. (2017). Spontaneous eye blink rate (EBR) is uncorrelated with dopamine D2 receptor availability and unmodulated by dopamine agonism in healthy adults. *eNeuro*, *4*(5).

Kenemans, J. L., Wieleman, J. S., Zeegers, M., & Verbaten, M. N. (1999). Caffeine and stroop interference. *Pharmacology Biochemistry and Behavior, 63*(4), 589-598.

Logemann, H. A., Böcker, K. B., Deschamps, P. K., van Harten, P. N., Koning, J., Kemner, C., . . . Kenemans, J. L. (2017). Haloperidol 2 mg impairs inhibition but not visuospatial attention. *Psychopharmacology, 234*(2), 235-244.

Rakshi, J. S., Uema, T., Ito, K., Bailey, D. L., Morrish, P. K., Ashburner, J., ... & Brooks, D. J. (1999). Frontal, midbrain and striatal dopaminergic function in early and advanced Parkinson's disease A 3D [18F] dopa-PET study. *Brain*, *122*(9), 1637-1650.

Sescousse, G., Ligneul, R., van Holst, R. J., Janssen, L. K., de Boer, F., Janssen, M., ... & Cools, R. (2018). Spontaneous eye blink rate and dopamine synthesis capacity: preliminary evidence for an absence of positive correlation. *European Journal of Neuroscience*, *47*(9), 1081-1086.

Wald, F. D., & Mellenbergh, G. J. (1990). De verkorte versie van de nederlandse vertaling van de profile of mood states (POMS). *Nederlands Tijdschrift Voor De Psychologie En Haar Grensgebieden,*
